# Preoperative Anemia or Low Hemoglobin Predicts Poor Prognosis in Gastric Cancer Patients: A Meta-Analysis

**DOI:** 10.1155/2019/7606128

**Published:** 2019-01-02

**Authors:** Xuan-zhang Huang, Yu-chong Yang, You Chen, Cong-cong Wu, Rui-fang Lin, Zhen-ning Wang, Xi Zhang

**Affiliations:** ^1^Department of Chemotherapy and Radiotherapy, The Second Affiliated Hospital and Yuying Children's Hospital of Wenzhou Medical University, 109 Xueyuan West Road, Lucheng District, Wenzhou City 325027, China; ^2^Tianjin Medical University Cancer Institute and Hospital, National Clinical Research Center for Cancer, Tianjin's Clinical Research Center for Cancer, Key Laboratory of Cancer Prevention and Therapy, Tianjin Medical University, Tianjin 300060, China; ^3^Wenzhou Dental Hospital, 197 Fuqian Street, Lucheng District, Wenzhou City 325027, China; ^4^Department of Surgical Oncology and General Surgery, The First Hospital of China Medical University, 155 North Nanjing Street, Heping District, Shenyang City 110001, China

## Abstract

**Background:**

The prognostic value of preoperative anemia in gastric cancer remains unclear. Therefore, the purpose of the present study is to evaluate the prognostic value of preoperative anemia in gastric cancer.

**Methods:**

We searched Embase and PubMed databases for relevant studies from inception to March 2018. The prognostic value of preoperative anemia in gastric cancer was determined by calculating the hazard ratio (HR) and the corresponding 95% confidence interval (CI) as effect measures. A random effect model was used in cases in which there was significant heterogeneity; otherwise, a fixed effect model was used. Statistical analyses were performed using Stata software.

**Results:**

Seventeen studies involving 13,154 gastric cancer patients were included. The estimated rate of preoperative anemia was 36% (95%CI = 27-44%). The overall survival of preoperative anemia was poor (HR = 1.33, 95%CI = 1.21-1.45). Moreover, disease-free survival was significantly lower in patients with preoperative anemia compared with those without this condition (HR = 1.62, 95%CI = 1.13-2.32). These findings were corroborated by the results of subgroup analyses.

**Conclusions:**

The results indicate that preoperative anemia predicts poor prognosis in gastric cancer, including overall survival and disease-free survival. Therefore, preoperative anemia may be a convenient and cost-effective blood-derived prognostic marker for gastric cancer.

## 1. Introduction

Gastric cancer is the fourth most frequently diagnosed cancer worldwide, accounting for approximately 951,600 new cases [1]. Surgical resection with adjuvant treatment is the main treatment for gastric cancer. However, despite improvements in the diagnosis and treatment of gastric cancer, the prognosis is still poor and gastric cancer remains the third leading cause of cancer-related death, accounting for approximately 723,100 deaths [1]. The tumor-node-metastasis staging system is recognized as an important prognostic factor in gastric cancer but does not provide complete prognostic information [2]. Therefore, identifying and characterizing other biomarkers are essential to improve prognosis.

Recently, blood-derived biomarkers have become attractive, convenient, and cost-effective prognostic markers to assess and predict the prognosis of gastric cancer. Several clinical studies have reported that the neutrophil-to-lymphocyte ratio, platelet-to-lymphocyte ratio, and monocyte-to-lymphocyte ratio are inversely correlated with the prognosis of gastric cancer [3–6]. Nevertheless, the prognostic value of preoperative anemia, which is a common hematological abnormality in gastric cancer, is controversial and has not been confirmed.

To date, no meta-analyses have evaluated the prognostic value of preoperative anemia in gastric cancer. Therefore, the objective of this study is to evaluate the relationship between preoperative anemia and the prognosis of gastric cancer using meta-analysis.

## 2. Materials and Methods

### 2.1. Literature Search

We systematically searched Embase and PubMed databases for relevant studies (up to March 2018). Furthermore, the reference lists of retrieved studies were manually searched for potentially eligible studies. The following keywords were used: “anemia,” “anaemia,” “hypohemia,” “hemoglobin,” “haemoglobin,” “hematocrystallin,” “gastric cancer,” “gastric tumor,” “gastric neoplasm,” “gastric carcinoma,” “stomach cancer,” “stomach tumor,” “stomach neoplasm,” “stomach carcinoma,” “prognosis,” “prognostic,” “survival,” “recurrence,” “relapse,” “mortality,” “risk,” and “outcome” (Supplementary File 1).

### 2.2. Inclusion Criteria

The studies that met the following criteria were included: (1) patients were diagnosed with gastric cancer, (2) patients were diagnosed with anemia before the operation, (3) the prognostic value of preoperative anemia in gastric cancer was evaluated, and (4) outcome measures were extracted directly or indirectly. In cases in which there were duplicate studies based on the same population, only the most informative study was included. Duplicate studies were identified by checking the baseline characteristics of each study.

### 2.3. Data Extraction and Quality Assessment

Two reviewers (Xuan-zhang Huang and Yu-chong Yang) independently extracted data and assessed study quality. The following data were extracted: first author, country of publication, year of publication, patient characteristics, cut-off value, rate of preoperative anemia, follow-up duration, and prognostic value (overall survival (OS) and disease-free survival (DFS)). The Newcastle-Ottawa Scale (NOS) criteria were used to assess the study quality [7]. Disagreements were resolved by comprehensive discussion.

### 2.4. Statistical Analysis

This meta-analysis was conducted following the Preferred Reporting Items for Systematic Reviews and Meta-analyses (PRISMA) statement (Supplementary File 2) [8]. The prognostic value of preoperative anemia in gastric cancer was determined by calculating the hazard ratio (HR) and the corresponding 95% confidence interval (CI). The HR and 95% CI values not available in the studies were calculated from the given data using the methods reported by Tierney et al. [9]. All relevant studies were included to conduct the overall analysis. Moreover, subgroup analyses were performed according to sample size, cut-off value, study quality, tumor stage, type of analysis, and country of publication.

Heterogeneity among the included studies was analyzed using the Cochran *Q* test and *I* ^2^ statistic and was considered statistically significant when the *p* value was <0.10 and/or *I* ^2^ was >50% [10]. A random effect model was used in cases in which heterogeneity was significant; otherwise, a fixed effect model was used. Metaregression analysis and subgroup analysis were conducted to determine potential sources of heterogeneity [11]. Begg's and Egger's tests and funnel plot were used to evaluate publication bias [12, 13]. Trim-and-fill analysis was performed to determine the impact of publication bias on the results [14].

A two-sided *p* < 0.05 was considered statistically significant. Statistical analyses were conducted using Stata software version 12.0 (Stata Corporation, College Station, TX, USA).

## 3. Results

### 3.1. Study Selection and Study Characteristics

**Figure 1:**
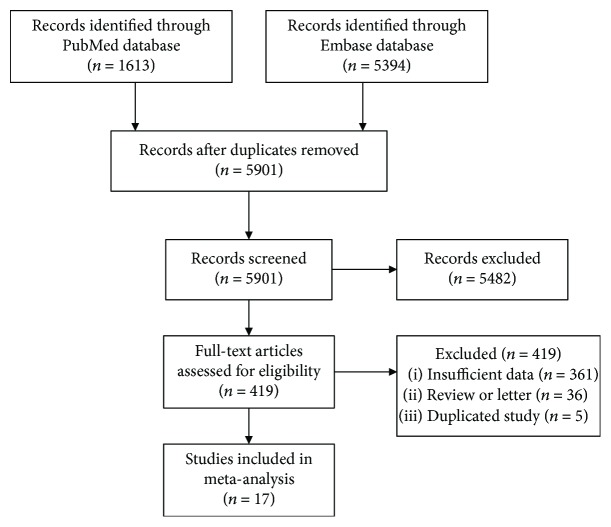
Literature search and study selection.

A total of 7,007 studies were initially identified from the literature search. After reviewing the titles and abstracts, 6,588 studies were excluded and 419 studies were further evaluated by a full-text review. Then, 402 studies were excluded after the full-text review because data were insufficient (361 studies) or they were reviews or letters (36 studies) or duplicates (5 studies). Therefore, 17 studies were eligible for this meta-analysis (Figure 1) [15–31].

Seventeen studies involving 13,154 patients were included, and the median sample size was 357 (range = 210–3012). The estimated rate of preoperative anemia was 36% (95%CI = 27-44%). The studies were from Italy, Czech Republic, Austria, China, Japan, Korea, and Thailand, and the year of publication ranged from 1995 to 2018. With respect to outcomes, eleven studies [15, 18, 21–23, 25–28, 30, 31] assessed the relationship between OS and preoperative anemia, one study [19] determined the relationship between DFS and preoperative anemia, and five studies [16, 17, 20, 24, 29] studied the relationship between OS, DFS, and preoperative anemia. Of the eligible studies, 14 studies [15–20, 22, 25–31] defined the cut-off value for preoperative anemia, and three studies [21, 23, 24] did not define this value. The baseline characteristics and study quality are summarized in Table 1.

### 3.2. Preoperative Anemia and OS

**Figure 2:**
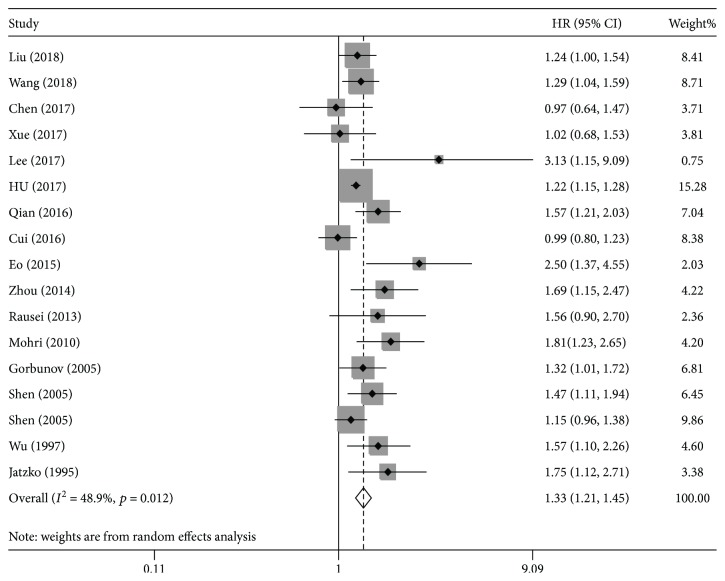
The hazard ratio (HR) was summarized for the relationship between preoperative anemia and overall survival.

Sixteen studies [15–18, 20–31] assessed the association between preoperative anemia and OS. Our results indicated that preoperative anemia predicted poor OS in gastric cancer (HR = 1.33, 95%CI = 1.21-1.45, *I* ^2^ = 48.9%; Figure 2). These findings were confirmed by subgroup analyses according to sample size and cut-off values (cut − off value ≥ 110: HR = 1.32, 95%CI = 1.21-1.44, *I* ^2^ = 28.4%; cut − off value < 110: HR = 1.48, 95%CI = 1.21-1.80, *I* ^2^ = 31.3%; sample size ≥ 500: HR = 1.27, 95%CI = 1.16-1.39, *I* ^2^ = 42.4%; sample size < 500: HR = 1.48, 95%CI = 1.18-1.86, *I* ^2^ = 50.8%). Moreover, the pooled result based on the multivariate analysis of HRs was significant (HR = 1.25, 95%CI = 1.13-1.39, *I* ^2^ = 45.9%). These results were confirmed by subgroup analysis stratified by tumor stage, study quality, and country of publication, indicating that preoperative anemia had poor OS (Table 2).

### 3.3. Preoperative Anemia and DFS

**Figure 3:**
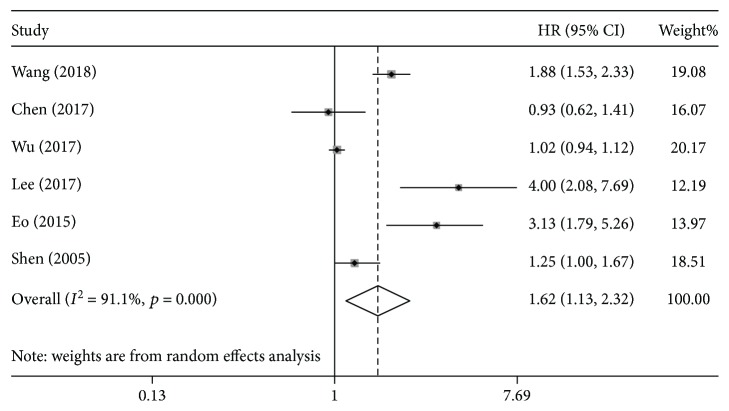
The hazard ratio (HR) was summarized for the relationship between preoperative anemia and disease-free survival.

HRs for DFS were available in six studies [16, 17, 19, 20, 24, 29]. The results indicated that the DFS was worse in patients with preoperative anemia (HR = 1.62, 95%CI = 1.13-2.32, *I* ^2^ = 91.1%; Figure 3). The results of subgroup analyses according to sample size, cut-off value, type of analysis, and country of publication indicated that the prognostic value of preoperative anemia on DFS was similar between sample size ≥ 500 (HR = 1.55, 95%CI = 1.03-2.31, *I* ^2^ = 83.1%), cut − off value ≥ 110 (HR = 1.62, 95%CI = 1.06-2.49, *I* ^2^ = 84.9%), univariate HR (HR = 2.07, 95%CI = 1.21-3.54, *I* ^2^ = 84.6%), and Asian countries (HR = 1.62, 95%CI = 1.13 − 2.32, *I* ^2^ = 91.1%) (Table 2). Moreover, preoperative anemia tended to have worse DFS when sample size was <500 (HR = 1.76, 95%CI = 0.94-3.26, *I* ^2^ = 90.6%). However, subgroup analyses were not performed for non-Asian countries because of the limited number of studies.

### 3.4. Publication Bias and Heterogeneity

Our results indicated that there was no significant publication bias, except in the overall analysis (*P*_Begg's_ = 0.023; *P*_Egger's_ = 0.028) and the subgroup analysis of sample size ≥ 500 on OS (*P*_Begg's_ = 0.048; *P*_Egger's_ = 0.223). In addition, publication bias did not affect the results for OS (HR = 1.22, 95%CI = 1.17–1.28 and HR = 1.21, 95%CI = 1.16–1.27, respectively). A metaregression analysis was conducted to identify potential sources of heterogeneity, and the results indicated that sample size, cut-off value, study quality, type of analysis, tumor stage, and country of publication were not significant sources of heterogeneity (Table 3).

## 4. Discussion

Preoperative anemia is common and multifactorial in malignancy patients [32, 33]. Several studies have demonstrated that bone marrow involvement, tumor-associated blood loss, cytokine-mediated disorder, and nutritional deficiencies in iron or folic acid play a vital role in the occurrence and maintenance of cancer-related anemia [34–36]. Studies reported that preoperative anemia was negatively correlated with quality of life and prognosis in patients with cancer [37–39]. Nevertheless, the clinical value of preoperative anemia in gastric cancer remains unclear. For this reason, this study assessed the relationship between preoperative anemia and the prognosis of gastric cancer.

Our study included 17 eligible studies involving 13,154 patients. The rate of preoperative anemia was 36% (95%CI = 27-44%). The overall results indicated that preoperative anemia was correlated with poor prognosis in gastric cancer (OS: HR = 1.33, 95%CI = 1.21-1.45; DFS: HR = 1.62, 95%CI = 1.13-2.32). These results were confirmed by subgroup analysis stratified by tumor stage, study quality, and country of publication. Therefore, preoperative anemia may be a useful biomarker to provide individualized treatment and improve clinical decision-making for gastric cancer before surgery.

The relationship between anemia and poor prognosis may be due to several factors. Tumor hypoxia and poor tumor oxygenation are common in advanced solid tumors and may increase the resistance to therapy because of an imbalance between oxygen supply and consumption [40, 41]. Anemia may intensify tumor hypoxia and poor tumor oxygenation and consequently decrease the effectiveness of therapy, especially when the hemoglobin level is <100 g/L [40]. Tumor hypoxia induces the expression and transcription of hypoxia-inducible factor-1 (HIF-1), which adaptively responds to hypoxia [40, 42]. HIF-1 activates many target genes, and HIF-1-regulated gene products (i.e., vascular endothelial growth factor, epidermal growth factor, erythropoietin, glucose transporters, and glycolytic enzymes) facilitate tumor survival, proliferation, invasion, and metastasis [42, 43]. In addition, several studies suggested that hypoxia might induce tumor aggressiveness and progression via genetic changes and clonal selection in tumor cells [43, 44]. Future studies are required to elucidate the mechanisms underlying the relationship between anemia and poor prognosis in gastric cancer.

Furthermore, the relationship between clinicopathological characteristics and preoperative anemia in gastric cancer is unclear. Among the evaluated studies, Shen et al. included 1688 gastric cancer patients and found that preoperative anemia was significantly associated with large tumor size, greater invasion depth, lymph node metastasis, and advanced tumor stages [29]. Liu et al. reported that preoperative anemia was associated with larger tumor size [15]. Therefore, preoperative anemia may be a potential biomarker for high tumor burden and aggressive tumor phenotype, and tumor-associated blood loss and malnutrition may occur in tumors with a large size and advanced stage [45]. In addition, several clinical studies reported that preoperative anemia was a significant risk factor for postoperative complications in gastric cancer and was inversely correlated with performance status and nutritional status [15, 29, 46, 47]. Nonetheless, pooled analysis to determine the relationship between these factors was not conducted because the number of studies was limited and the underlying mechanism of this relationship was unclear. Future studies are required to evaluate the association between tumor-related and patient-related factors and preoperative anemia to provide additional prognostic and tumor information.

Preoperative anemia may be helpful to tailor individual treatment. Gastric cancer patients without anemia may better tolerate the toxicity of surgery and adjuvant therapy, and patients with anemia need to be treated before surgery and adjuvant therapy, with close follow-up. In clinical practice, blood transfusion has been widely used for correcting anemia during surgery. However, accumulating evidence indicates that perioperative blood transfusion cannot improve prognosis in gastric cancer patients [19, 25, 48, 49]. In this respect, Sun et al. performed a pooled analysis of 18 studies with 9120 gastric cancer patients and found that perioperative blood transfusion was strongly correlated with all-cause mortality, cancer-related mortality, and recurrence [50]. Furthermore, patients with perioperative blood transfusion had more postoperative complications than those without blood transfusion [48, 51]. Erythropoiesis-stimulating agents can be used to treat anemia; however, it is unknown whether these agents improve prognosis, and experimental studies indicate that erythropoietin may induce the expression of the vascular endothelial growth factor and stimulate tumor recurrence in mice. Therefore, high-quality, large-scale clinical trials are required to identify beneficial intervention strategies that may not adversely affect prognosis for anemia treatment in patients with gastric cancer.

This study has several limitations. First, our meta-analysis was not registered online. However, the literature search strategy and the inclusion and exclusion criteria were defined prospectively to prevent potential biases. Second, our meta-analysis was performed using published data from the included studies and detailed individual data on tumor characteristics, patient characteristics, treatment strategies, and follow-up time were not obtained. Third, anemia was disease status rather than an intervention measure and consequently could not be manually intervened or randomized in clinical trials. Therefore, the included studies were cohort studies rather than randomized controlled trials. However, a larger sample size might provide an effective basis for predicting the prognostic value of preoperative anemia in gastric cancer. Moreover, there was a considerable degree of heterogeneity among the included studies, and our metaregression analysis found no significant sources of heterogeneity (Table 3). Heterogeneity was not significantly decreased in the subgroup analysis stratified by sample size, cut-off value, study quality, type of analysis, tumor stage, and country of publication, confirming the results of the metaregression analysis. The unexplained heterogeneity may be caused by differences in patient characteristics (i.e., age, gender, and race), treatment strategies, diagnostic methods, and other confounding factors. Therefore, heterogeneity could not be completely removed and may underestimate the prognostic value of preoperative anemia in gastric cancer because a relatively conservative random effect model was used to pool the results. Further large-scale multicenter studies on homogeneous populations and treatment strategies are required to determine this prognostic value. In addition, the limited number of subgroup analyses might affect the statistical power of the results.

## 5. Conclusion

Our results indicate that preoperative anemia predicts poor prognosis in gastric cancer, including overall survival and disease-free survival. Therefore, preoperative anemia is a convenient and cost-effective blood-derived biomarker for predicting prognosis in gastric cancer. Further well-designed, high-quality, large-scale multicenter studies are needed to evaluate whether the individualized treatment of preoperative anemia can improve the prognosis of gastric cancer.

## Figures and Tables

**Table 1:** The baseline characteristics and design variables of included studies on preoperative anemia and gastric cancer prognosis.

Article	Country	Year	Study type	Number of patients (M/F)^a^	Age: mean ± SD/median (range)^b^	Rate of anemia (*n*/*N*)^c^	Follow-up: mean ± SD/median (range)^d^	Outcome measured	Study quality
Liu	China	2018	Cohort	2163 (1442/721)	Median: 58 (14-89)	314/1035	Median: 28 (3-185)	OS	7
Wang	China	2018	Cohort	859 (672/187)	Median: 64 (IQR: 16)	434/859	Median: 60.9	DFS, OS	6
Chen	China	2017	Cohort	292 (205/87)	Mean: 57 (range: 28-77)	144/292	NR	DFS, OS	6
Xue	China	2017	Cohort	269 (202/67)	Mean: 65.77 (95% CI: 64.42-67.12)	84/269	Median: 40 (1-108)	OS	8
Wu	China	2017	Cohort	210 (128/82)	Median: 60 (36-79)	53/210	60	DFS	8
Lee	Korea	2017	Cohort	309 (77/232)	Median: 60 (29-82)	NR	Median: 33.8 (2.6-67.5)	DFS, OS	6
Hu	China	2017	Cohort	3012 (2239/773)	Range: 51-68	NR	Median: 44.05 (1.1-183.3)	OS	6
Qian	China	2016	Cohort	579 (405/174)	Median: 58 (18-85)	252/579	Median: 44 (12-81)	OS	7
Cui	China	2016	Cohort	1150 (817/333)	Range: 23-93	NR	Median: 40	OS	6
Eo	Korea	2015	Cohort	299 (195/104)	Median: 59 (25-92)	NR	Median: 37.2 (1.7-91.4)	DFS, OS	8
Zhou	China	2014	Cohort	605 (405/200)	Mean: 58.15 ± 11.39	90/605	Median: 35.4 (1-78)	OS	7
Rausei	Italy	2013	Cohort	224 (147/77)	Mean: 67 ± 11.6	111/224	Median: 48	OS	5
Mohri	Japan	2010	Cohort	357 (245/112)	Mean: 63.4 (range: 32-87)	NR	Median: 68 (1-70)	OS	6
Gorbunov	Czech Republic	2005	Cohort	283 (182/101)	NR	179/283	Mean: 73.2 (range: 12-132)	OS	6
Shen	Korea	2005	Cohort	1688 (1162/526)	Mean: 54.7 ± 11.8 (range: 22-85)	673/1688	Median: 85 (range: 0-138)	DFS, OS	8
Wu	Taiwan	1997	Cohort	510 (420/90)	NR	84/510	NR	OS	6
Jatzko	Austria	1995	Cohort	345 (221/124)	Mean: 67 ± 0.57	45/339	Mean: 65.5	OS	7

^a^M/F presents the number of males and females, respectively. ^b^The age of patients was summarized as mean with standard deviation or median with range. ^c^The rate of patients with preoperative anemia. ^d^The follow-up period was summarized as mean with standard deviation or median with range. Abbreviations: DFS: disease-free survival; IQR: interquartile range; M/F: males/females; NR: not reported; OS: overall survival; SD: standard deviation.

**Table 2:** The results of subgroup analyses for the prognostic value of preoperative anemia.

	HR	95% CI	*P*	*I* ^2^ (%)	Publication bias
*Overall survival*					
Overall	1.33	1.21-1.45	<0.01	48.90%	Begg′s test = 0.023; Egger′s test = 0.028
Sample size					
≥500	1.27	1.16-1.39	<0.01	42.40%	Begg′s test = 0.048; Egger′s test = 0.223
<500	1.48	1.18-1.86	<0.01	50.80%	Begg′s test = 0.266; Egger′s test = 0.174
Cut-off point					
≥110	1.32	1.21-1.44	<0.01	28.40%	Begg′s test = 0.107; Egger′s test = 0.085
<110	1.48	1.21-1.80	<0.01	31.30%	Begg′s test = 0.734; Egger′s test = 0.881
Study quality					
≥7	1.40	1.20-1.63	<0.01	49.30%	Begg′s test = 0.174; Egger′s test = 0.070
<7	1.28	1.13-1.44	<0.01	46.80%	Begg′s test = 0.118; Egger′s test = 0.254
Study analysis type					
Multivariate type	1.25	1.13-1.39	<0.01	45.90%	Begg′s test = 0.536; Egger′s test = 0.427
Univariate type	1.65	1.39-1.95	<0.01	71.60%	Begg′s test = 0.200; Egger′s test = 0.170
Tumor stage					
I–II	1.33	1.04-1.71	0.03	20.00%	Begg′s test = 1.000; Egger′s test = 0.469
III	1.24	0.68-2.26	0.49	77.10%	Begg′s test = 1.000; Egger′s test = /
Country					
Asia	1.31	1.18-1.45	<0.01	53.90%	Begg′s test = 0.080; Egger′s test = 0.087
Non-Asia	1.44	1.17-1.78	<0.01	0.00%	Begg′s test = 1.000; Egger′s test = 0.374
*Disease-free survival*					
Overall	1.62	1.13-2.32	<0.01	91.10%	Begg′s test = 0.452; Egger′s test = 0.097
Sample size					
≥500	1.55	1.03-2.31	0.03	83.10%	Begg′s test = 1.000; Egger′s test = /
<500	1.76	0.94-3.26	0.07	90.60%	Begg′s test = 0.089; Egger′s test = 0.216
Cut-off point					
≥110	1.62	1.06-2.49	0.03	84.90%	Begg′s test = 1.000; Egger′s test = 0.875
Study analysis type					
Multivariable type	1.09	0.91-1.30	0.35	51.60%	Begg′s test = 1.000; Egger′s test = /
Univariable type	2.07	1.21-3.54	<0.01	84.60%	Begg′s test = 0.308; Egger′s test = 0.704
Country					
Asia	1.62	1.13-2.32	<0.01	91.10%	Begg′s test = 0.452; Egger′s test = 0.097

Abbreviations: CI: confidence interval; HR: hazard ratio; *I* ^2^: degree of heterogeneity; *P*: *P* for the HR; “/”: not applicable due to limited number of studies.

**Table 3:** Metaregression analysis exploring sources of heterogeneity.

	Coefficient	Standard error	*p*	Adjusted *R* ^2^
*Overall survival*				
Sample size	-0.00008	0.00005	0.177	6.65%
Cut-off value	-0.10590	0.13030	0.432	20.55%
Study quality	0.01575	0.06565	0.814	-23.34%
Study analysis type	-0.13012	0.10638	0.24	-2.74%
Tumor stage	-0.04170	0.04517	0.371	-16.14%
Country	-0.11614	0.16179	0.484	-2.02%
*Disease-free survival*				
Sample size	-0.00017	0.00049	0.74	-31.61%
Cut-off value	0.47822	0.63443	0.506	-20.93%
Study quality	-0.09480	0.26281	0.737	-27.41%
Study analysis type	-0.60512	0.45102	0.251	17.41%
Tumor stage	-0.69009	0.62650	0.333	1.19%

Note: the dependent variable is the lnHR for overall survival or disease-free survival from each study; weights have been assigned according to the estimated variance of the lnHR.

## Supplementary Materials

Supplementary 1 Detailed search strategies for databases.

Supplementary 2 PRISMA checklist.
