# Supplementary material for: Preoperative Anemia or Low Hemoglobin Predicts Poor Prognosis in Gastric Cancer Patients: A Meta-Analysis
Source: Dis Markers. 2019 Jan 2;2019:7606128. doi: 10.1155/2019/7606128 (PMC6334363; doi:10.1155/2019/7606128)
Supplement: Supplementary 1 — Detailed search strategies for databases. [file 7606128.f1.doc]

**Literature search (up to March 2018)**

**Databases and search strategies**

**PubMed**

(anemia OR anaemia OR hypohemia OR hemoglobin OR haemoglobin OR hematocrystallin) AND (gastric cancer OR stomach cancer OR gastric tumor OR stomach tumor OR gastric neoplasm OR stomach neoplasm OR gastric carcinoma OR stomach carcinoma) AND (prognosis OR prognostic OR survival OR recurrence OR relapse OR mortality OR risk OR outcome)

**Embase**

(anemia OR anaemia OR hypohemia OR hemoglobin OR haemoglobin OR hematocrystallin ) AND (gastric AND cancer OR (stomach AND cancer) OR (gastric AND tumor) OR (stomach AND tumor) OR (gastric AND neoplasm) OR (stomach AND neoplasm) OR (gastric AND carcinoma) OR (stomach AND carcinoma)) AND (prognosis OR prognostic OR survival OR recurrence OR relapse OR mortality OR risk OR outcome)
